# 5,17-Diformyl-25,26,27,28-tetra­propoxy­calix[4]arene

**DOI:** 10.1107/S1600536813004625

**Published:** 2013-02-23

**Authors:** Zhengyi Li, Hongzhao Ma, Yuan Lai, Liang Chen, Xiaoqiang Sun

**Affiliations:** a Jiangsu Key Laboratory of Fine Petrochemical Engineering, School of Petrochemical Engineering, Changzhou University, Changzhou 213164, Jiangsu, People’s Republic of China

## Abstract

The title compound, C_42_H_48_O_6_, was obtained *via* formyl­ation of 25,26,27,28-tetra­propoxycalix[4]arene with dichloro­methyl methyl ether and tin tetra­chloride. It adopts a pinched cone conformation, which leads to an open cavity. The two opposite aromatic rings bearing formyl groups are almost parallel, making a dihedral angle of 29.1 (2)°. The other pair of opposite rings are close to being perpendicular, making a dihedral angle of 73.6 (1)°. Adjacent rings are almost perpendicular, making dihedral angles of 78.8 (2), 81.6 (1), 78.2 (1) and 74.7 (1)°.

## Related literature

For general background to calix[4]arenes, see: Arduini *et al.* (1995 ▶); Decken *et al.* (2004 ▶); Seigle-Ferrand *et al.* (2006 ▶); Kennedy *et al.* (2010 ▶).
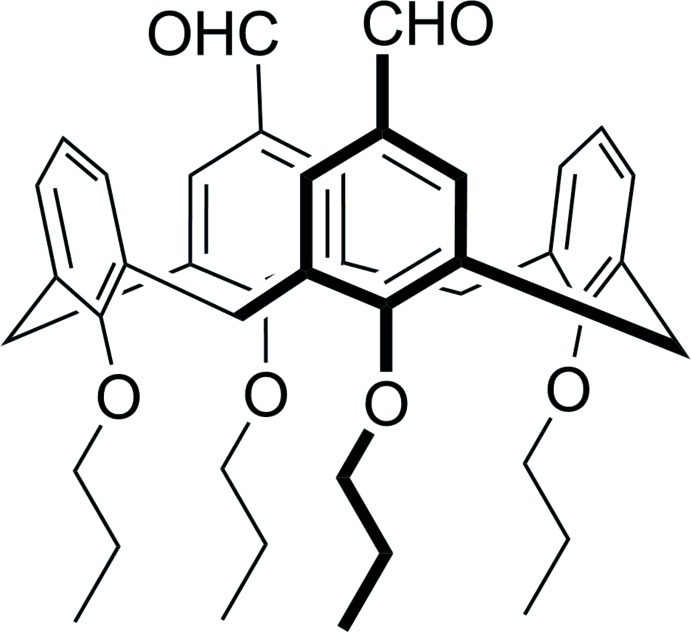


## Experimental

### 

#### Crystal data

C_42_H_48_O_6_
*M* *_r_* = 648.80
Orthorhombic, 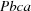

*a* = 14.7121 (15) Å
*b* = 17.5133 (19) Å
*c* = 28.912 (3) Å
*V* = 7449.5 (14) Å^3^
*Z* = 8
Mo *K*α radiation
μ = 0.08 mm^−1^
*T* = 296 K
0.20 × 0.18 × 0.15 mm

#### Data collection

Bruker SMART CCD area-detector diffractometer
Absorption correction: multi-scan (*SADABS*; Bruker, 2000 ▶) *T*_min_ = 0.985, *T*_max_ = 0.989
41325 measured reflections
6924 independent reflections
3908 reflections with *I* > 2σ(*I*)
*R*_int_ = 0.085

#### Refinement

*R*[*F* ^2^ > 2σ(*F* ^2^)] = 0.066
*wR*(*F* ^2^) = 0.220
*S* = 1.00
6924 reflections
437 parameters
1 restraint
H-atom parameters constrained
Δρ_max_ = 0.34 e Å^−3^
Δρ_min_ = −0.22 e Å^−3^

### 

Data collection: *SMART* (Bruker, 2000 ▶); cell refinement: *SAINT* (Bruker, 2000 ▶); data reduction: *SAINT*; program(s) used to solve structure: *SHELXTL* (Sheldrick, 2008 ▶); program(s) used to refine structure: *SHELXTL*; molecular graphics: *SHELXTL*; software used to prepare material for publication: *SHELXTL*.

## Supplementary Material

Crystal structure: contains datablock(s) I, global. DOI: 10.1107/S1600536813004625/fj2615sup1.cif

Structure factors: contains datablock(s) I. DOI: 10.1107/S1600536813004625/fj2615Isup2.hkl

Additional supplementary materials:  crystallographic information; 3D view; checkCIF report

## Figures and Tables

**Fig. 1.**
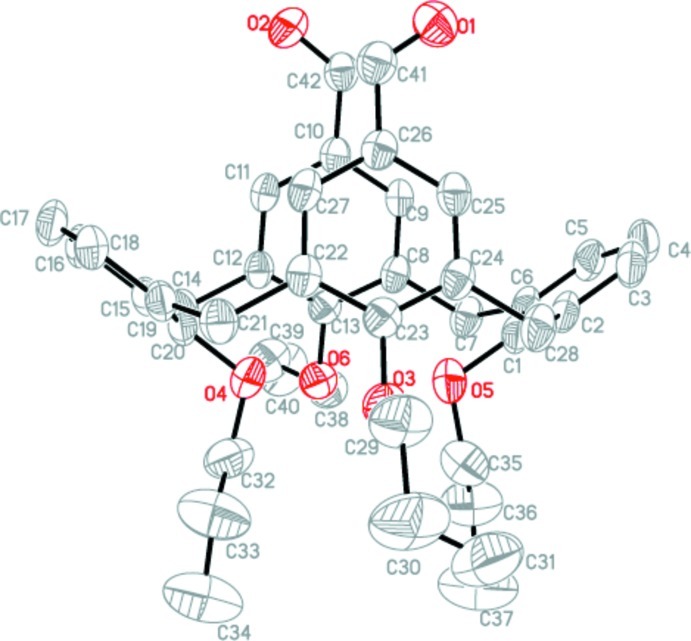
The molecular structure of the title compound. Displacement ellipsoids are drawn at the 30% probability level. All H atoms have been omitted for clarity.

## supplementary crystallographic information

### Comment

Regioselective formylation at the upper rim of calixarenes is an important way to obtain functional calixarene because aldehyde group can be easily transformed into hydroxy and carboxyl which are very useful in organic synthesis (Arduini *et al.*, 1995) and the suparamolecular self-assembly (Kennedy *et al.*, 2010). Although the crystal structures of 5,17-diformyl-25,27-dipropoxycalix[4]arene (Seigle-Ferrand *et al.*, 2006) and 5,17-Diformyl-25,26,27,28-tetrabenzyloxycalix[4]arene (Decken *et al.*, 2004) have been reported, we herein present the structure of 5,17-diformyl-25,26,27,28-tetrapropoxycalix[4]arene (Fig. 1).

The title compound adopts a pinched cone conformation with a small cavity. All phenyl rings are tilted to from the calix cavity, as defined by the angles which the aromatic rings make with the plane of the four bridging CH_2_ moieties (C7, C14, C21 and C28) which link them, *viz*. 36.8 (1)° (C1–C6 or C15–C20), 77.2 (1)° (C8–C13) and 73.5 (2)° (C22–C27), respectively. Two opposite aromatic rings (C8–C13 and C22–C27) with formyl groups are almost parallel [dihedral angle 29.1 (2)°], while the other pair of opposite rings (C1–C6 and C15–20) are close to being perpendicular to one another [dihedral angle 73.6 (1)°], and adjacent phenyl rings are almost perpendicular [dihedral angles 78.8 (2)°, 81.6 (1)°, 78.2 (1)° and 74.7 (1)°, respectively].

### Experimental

To a solution of 25,26,27,28-tetrapropoxycalix[4]arene (0.5 g, 0.84 mmol) in dry CHCl_3_ (35 ml) cooled at -35 °C were added 1,1-dichlorodimethyl ether (0.15 ml, 1.68 mmol) and tin tetrachloride (0.19 ml, 1.68 mmol). The reaction mixture was stirred at -35 °C for 5 min and then treated with water (50 ml). The organic layer was washed twice with water and dried by Na_2_SO_4_, and the solvent was evaporated under reduced pressure. Purification by column chromatography (petroleum ether/ethyl acetate 5:1) afforded white solid (0.23 g, yield 42.2%, m.p. > 573 K). Single crystals suitable for X-ray diffraction were obtained by evaporation of an enthanol solution.

### Refinement

All the H atoms were placed in geometrically idealized positions and constrained to ride on their parent atoms, with C—H distances of 0.93–0.97 Å, and with *U*_iso_(H) = 1.2*U*_eq_(C).

### Crystal data

**Table:**

C_42_H_48_O_6_	*F*(000) = 2784
*M**_r_* = 648.80	*D*_x_ = 1.157 Mg m^−^^3^
Orthorhombic, *P**b**c**a*	Mo *K*α radiation, λ = 0.71073 Å
Hall symbol: -P 2ac 2ab	Cell parameters from 6648 reflections
*a* = 14.7121 (15) Å	θ = 2.3–21.5°
*b* = 17.5133 (19) Å	µ = 0.08 mm^−^^1^
*c* = 28.912 (3) Å	*T* = 296 K
*V* = 7449.5 (14) Å^3^	Block, colourless
*Z* = 8	0.20 × 0.18 × 0.15 mm

### Data collection

**Table:**

Bruker SMART CCD area-detector diffractometer	6924 independent reflections
Radiation source: fine-focus sealed tube	3908 reflections with *I* > 2σ(*I*)
Graphite monochromator	*R*_int_ = 0.085
phi and ω scans	θ_max_ = 25.5°, θ_min_ = 1.9°
Absorption correction: multi-scan (*SADABS*; Bruker, 2000)	*h* = −17→13
*T*_min_ = 0.985, *T*_max_ = 0.989	*k* = −21→21
41325 measured reflections	*l* = −31→35

### Refinement

**Table:**

Refinement on *F*^2^	Primary atom site location: structure-invariant direct methods
Least-squares matrix: full	Secondary atom site location: difference Fourier map
*R*[*F*^2^ > 2σ(*F*^2^)] = 0.066	Hydrogen site location: inferred from neighbouring sites
*wR*(*F*^2^) = 0.220	H-atom parameters constrained
*S* = 1.00	*w* = 1/[σ^2^(*F*_o_^2^) + (0.1343*P*)^2^ + 0.050*P*] where *P* = (*F*_o_^2^ + 2*F*_c_^2^)/3
6924 reflections	(Δ/σ)_max_ < 0.001
437 parameters	Δρ_max_ = 0.34 e Å^−^^3^
1 restraint	Δρ_min_ = −0.22 e Å^−^^3^

### Special details

**Table:**

Geometry. All e.s.d.'s (except the e.s.d. in the dihedral angle between two l.s. planes) are estimated using the full covariance matrix. The cell e.s.d.'s are taken into account individually in the estimation of e.s.d.'s in distances, angles and torsion angles; correlations between e.s.d.'s in cell parameters are only used when they are defined by crystal symmetry. An approximate (isotropic) treatment of cell e.s.d.'s is used for estimating e.s.d.'s involving l.s. planes.
Refinement. Refinement of *F*^2^ against ALL reflections. The weighted *R*-factor *wR* and goodness of fit *S* are based on *F*^2^, conventional *R*-factors *R* are based on *F*, with *F* set to zero for negative *F*^2^. The threshold expression of *F*^2^ > σ(*F*^2^) is used only for calculating *R*-factors(gt) *etc*. and is not relevant to the choice of reflections for refinement. *R*-factors based on *F*^2^ are statistically about twice as large as those based on *F*, and *R*- factors based on ALL data will be even larger.

### Fractional atomic coordinates and isotropic or equivalent isotropic displacement parameters (Å^2^)

**Table:**

	*x*	*y*	*z*	*U*_iso_*/*U*_eq_	
O6	0.75730 (12)	0.16233 (10)	0.59545 (6)	0.0746 (5)	
O4	0.98908 (12)	0.13116 (9)	0.59448 (6)	0.0711 (5)	
O5	0.91208 (13)	0.31123 (10)	0.61795 (7)	0.0767 (5)	
O3	1.14504 (13)	0.28445 (10)	0.60310 (7)	0.0763 (5)	
O2	0.86628 (16)	0.06906 (13)	0.80074 (8)	0.1001 (7)	
C1	0.88905 (18)	0.36103 (13)	0.65298 (9)	0.0620 (6)	
C11	0.84777 (15)	0.08053 (13)	0.70120 (9)	0.0592 (6)	
H11	0.8748	0.0356	0.7114	0.071*	
C10	0.82985 (15)	0.13841 (13)	0.73289 (9)	0.0566 (6)	
C12	0.82641 (15)	0.08821 (13)	0.65480 (9)	0.0572 (6)	
C26	1.07862 (15)	0.21843 (14)	0.73661 (9)	0.0614 (6)	
C25	1.06269 (16)	0.29248 (14)	0.72202 (9)	0.0639 (7)	
H25	1.0380	0.3275	0.7426	0.077*	
C24	1.08283 (16)	0.31549 (13)	0.67726 (9)	0.0605 (6)	
C9	0.79508 (15)	0.20714 (12)	0.71674 (9)	0.0573 (6)	
H9	0.7875	0.2474	0.7373	0.069*	
C13	0.78363 (15)	0.15595 (13)	0.64102 (9)	0.0580 (6)	
C23	1.12262 (16)	0.26208 (14)	0.64775 (9)	0.0609 (6)	
C6	0.80146 (18)	0.35706 (13)	0.67044 (10)	0.0656 (7)	
C27	1.11350 (16)	0.16595 (14)	0.70534 (9)	0.0634 (7)	
H27	1.1218	0.1157	0.7148	0.076*	
O1	1.02457 (15)	0.23488 (13)	0.81322 (7)	0.0954 (6)	
C20	1.01733 (18)	0.06375 (13)	0.61494 (8)	0.0602 (6)	
C2	0.95571 (18)	0.40740 (13)	0.67259 (10)	0.0658 (7)	
C22	1.13630 (15)	0.18610 (14)	0.66045 (9)	0.0598 (6)	
C8	0.77141 (15)	0.21727 (13)	0.67091 (9)	0.0590 (6)	
C14	0.85238 (18)	0.02586 (14)	0.62104 (9)	0.0697 (7)	
H14A	0.8172	−0.0198	0.6274	0.084*	
H14B	0.8390	0.0422	0.5897	0.084*	
C42	0.84557 (16)	0.12809 (16)	0.78227 (10)	0.0681 (7)	
H42	0.8390	0.1708	0.8011	0.082*	
C15	0.95255 (17)	0.00841 (13)	0.62548 (9)	0.0631 (7)	
C7	0.73768 (17)	0.29499 (14)	0.65468 (10)	0.0719 (7)	
H7A	0.7336	0.2954	0.6212	0.086*	
H7B	0.6774	0.3044	0.6671	0.086*	
C28	1.05459 (18)	0.39427 (14)	0.66110 (11)	0.0760 (8)	
H28A	1.0917	0.4327	0.6762	0.091*	
H28B	1.0637	0.3986	0.6280	0.091*	
C21	1.16861 (17)	0.12626 (15)	0.62629 (10)	0.0706 (7)	
H21A	1.1682	0.1474	0.5953	0.085*	
H21B	1.2304	0.1114	0.6337	0.085*	
C19	1.10750 (18)	0.05747 (14)	0.62828 (9)	0.0636 (7)	
C18	1.1346 (2)	−0.01116 (16)	0.64855 (10)	0.0752 (8)	
H18	1.1951	−0.0178	0.6571	0.090*	
C3	0.9293 (2)	0.45609 (14)	0.70747 (11)	0.0797 (8)	
H3	0.9720	0.4886	0.7206	0.096*	
C5	0.7783 (2)	0.40644 (15)	0.70581 (10)	0.0775 (8)	
H5	0.7198	0.4052	0.7180	0.093*	
C16	0.9842 (2)	−0.05922 (14)	0.64578 (10)	0.0772 (8)	
H16	0.9433	−0.0982	0.6523	0.093*	
C17	1.0738 (2)	−0.06872 (16)	0.65606 (11)	0.0844 (9)	
H17	1.0938	−0.1149	0.6683	0.101*	
C4	0.8413 (2)	0.45786 (16)	0.72333 (11)	0.0860 (9)	
H4	0.8241	0.4933	0.7457	0.103*	
C41	1.05586 (19)	0.19441 (17)	0.78352 (11)	0.0760 (8)	
H41	1.0664	0.1436	0.7911	0.091*	
C38	0.6601 (2)	0.15937 (19)	0.58853 (11)	0.0884 (9)	
H38A	0.6466	0.1754	0.5572	0.106*	
H38B	0.6314	0.1954	0.6094	0.106*	
C33	1.0501 (4)	0.1401 (4)	0.51739 (16)	0.173 (2)	
H33A	1.0788	0.1872	0.5273	0.207*	
H33B	1.0928	0.0989	0.5230	0.207*	
C32	0.9706 (3)	0.1274 (2)	0.54593 (13)	0.1158 (12)	
H32A	0.9249	0.1653	0.5383	0.139*	
H32B	0.9455	0.0776	0.5388	0.139*	
C35	0.9231 (3)	0.3447 (2)	0.57389 (13)	0.1139 (12)	
H35A	0.9649	0.3139	0.5558	0.137*	
H35B	0.9498	0.3950	0.5775	0.137*	
C39	0.6201 (2)	0.0831 (2)	0.59624 (14)	0.1087 (11)	
H39A	0.6340	0.0662	0.6274	0.130*	
H39B	0.6470	0.0469	0.5748	0.130*	
C37	0.8426 (4)	0.3775 (5)	0.50134 (19)	0.215 (3)	
H37A	0.8726	0.4262	0.5008	0.323*	
H37B	0.7832	0.3824	0.4879	0.323*	
H37C	0.8775	0.3413	0.4839	0.323*	
C40	0.5179 (3)	0.0840 (3)	0.58954 (17)	0.1420 (16)	
H40A	0.4911	0.1204	0.6103	0.213*	
H40B	0.4937	0.0342	0.5959	0.213*	
H40C	0.5040	0.0979	0.5582	0.213*	
C29	1.2359 (3)	0.3127 (2)	0.59968 (13)	0.1179 (13)	
H29A	1.2786	0.2723	0.6070	0.142*	
H29B	1.2448	0.3539	0.6217	0.142*	
C36	0.8345 (3)	0.3518 (4)	0.54810 (17)	0.1536 (18)	
H36A	0.8047	0.3024	0.5481	0.184*	
H36B	0.7955	0.3871	0.5647	0.184*	
C34	1.0344 (4)	0.1447 (4)	0.46906 (16)	0.207 (3)	
H34A	0.9974	0.1024	0.4595	0.311*	
H34B	1.0914	0.1431	0.4530	0.311*	
H34C	1.0037	0.1917	0.4621	0.311*	
C31	1.2117 (6)	0.4091 (4)	0.5406 (2)	0.224 (3)	
H31A	1.1523	0.3992	0.5282	0.337*	
H31B	1.2478	0.4352	0.5179	0.337*	
H31C	1.2063	0.4403	0.5677	0.337*	
C30	1.2528 (4)	0.3410 (4)	0.55198 (19)	0.178 (2)	
H30A	1.3179	0.3468	0.5479	0.214*	
H30B	1.2324	0.3023	0.5303	0.214*	

### Atomic displacement parameters (Å^2^)

**Table:**

	*U*^11^	*U*^22^	*U*^33^	*U*^12^	*U*^13^	*U*^23^
O6	0.0729 (12)	0.0742 (12)	0.0766 (12)	−0.0064 (9)	−0.0042 (9)	0.0019 (10)
O4	0.0751 (12)	0.0508 (10)	0.0873 (13)	0.0007 (8)	0.0014 (9)	0.0081 (9)
O5	0.0787 (13)	0.0567 (11)	0.0945 (14)	0.0011 (9)	−0.0069 (10)	−0.0133 (10)
O3	0.0785 (12)	0.0700 (12)	0.0805 (13)	−0.0166 (9)	−0.0020 (9)	0.0046 (10)
O2	0.1230 (18)	0.0842 (15)	0.0930 (15)	0.0323 (13)	−0.0075 (12)	0.0146 (12)
C1	0.0727 (17)	0.0399 (12)	0.0733 (16)	0.0093 (11)	−0.0103 (13)	−0.0059 (12)
C11	0.0534 (14)	0.0419 (12)	0.0823 (18)	−0.0012 (10)	0.0040 (12)	0.0014 (12)
C10	0.0467 (13)	0.0506 (14)	0.0724 (16)	0.0019 (10)	0.0015 (11)	−0.0018 (12)
C12	0.0516 (13)	0.0436 (13)	0.0763 (17)	−0.0072 (10)	0.0053 (11)	−0.0038 (12)
C26	0.0529 (14)	0.0570 (15)	0.0744 (16)	0.0010 (11)	−0.0077 (12)	0.0006 (13)
C25	0.0556 (14)	0.0537 (15)	0.0825 (18)	−0.0016 (11)	−0.0064 (12)	−0.0102 (13)
C24	0.0542 (14)	0.0472 (14)	0.0802 (17)	−0.0067 (11)	−0.0140 (12)	0.0006 (12)
C9	0.0526 (13)	0.0431 (13)	0.0762 (17)	0.0008 (10)	0.0005 (11)	−0.0075 (12)
C13	0.0545 (14)	0.0538 (14)	0.0656 (16)	−0.0089 (11)	−0.0024 (11)	0.0026 (12)
C23	0.0536 (14)	0.0595 (15)	0.0697 (16)	−0.0120 (11)	−0.0071 (12)	−0.0013 (13)
C6	0.0715 (17)	0.0421 (13)	0.0832 (18)	0.0125 (12)	−0.0155 (14)	0.0021 (12)
C27	0.0570 (15)	0.0491 (14)	0.0841 (18)	0.0059 (11)	−0.0073 (12)	0.0021 (13)
O1	0.1027 (16)	0.0954 (16)	0.0880 (14)	0.0065 (12)	0.0118 (12)	−0.0073 (13)
C20	0.0727 (17)	0.0428 (13)	0.0649 (15)	0.0022 (12)	0.0119 (12)	−0.0082 (11)
C2	0.0771 (17)	0.0356 (12)	0.0848 (18)	0.0024 (11)	−0.0151 (13)	0.0014 (12)
C22	0.0493 (13)	0.0527 (14)	0.0774 (17)	−0.0021 (10)	−0.0044 (11)	−0.0036 (13)
C8	0.0481 (13)	0.0452 (13)	0.0837 (18)	−0.0015 (10)	−0.0044 (11)	0.0018 (12)
C14	0.0741 (17)	0.0510 (14)	0.0840 (18)	−0.0116 (12)	0.0090 (13)	−0.0123 (13)
C42	0.0585 (15)	0.0652 (17)	0.0806 (18)	0.0095 (12)	−0.0005 (13)	−0.0045 (14)
C15	0.0722 (17)	0.0449 (13)	0.0722 (16)	0.0002 (12)	0.0131 (12)	−0.0099 (12)
C7	0.0595 (15)	0.0566 (15)	0.099 (2)	0.0061 (12)	−0.0153 (13)	0.0031 (14)
C28	0.0765 (18)	0.0500 (15)	0.102 (2)	−0.0113 (13)	−0.0134 (15)	−0.0018 (14)
C21	0.0599 (16)	0.0685 (17)	0.0834 (18)	0.0052 (12)	0.0054 (13)	−0.0056 (14)
C19	0.0701 (17)	0.0505 (14)	0.0702 (16)	0.0068 (12)	0.0108 (12)	−0.0087 (12)
C18	0.0794 (18)	0.0635 (17)	0.0826 (19)	0.0203 (15)	0.0069 (14)	−0.0080 (15)
C3	0.092 (2)	0.0448 (14)	0.102 (2)	0.0049 (14)	−0.0246 (17)	−0.0098 (15)
C5	0.0831 (19)	0.0543 (16)	0.095 (2)	0.0209 (14)	0.0003 (15)	0.0026 (15)
C16	0.097 (2)	0.0399 (14)	0.094 (2)	−0.0013 (13)	0.0215 (16)	−0.0024 (13)
C17	0.102 (2)	0.0497 (16)	0.101 (2)	0.0205 (16)	0.0168 (18)	0.0045 (15)
C4	0.111 (3)	0.0561 (17)	0.091 (2)	0.0193 (17)	−0.0109 (18)	−0.0174 (15)
C41	0.0706 (18)	0.0704 (18)	0.087 (2)	0.0004 (14)	−0.0032 (15)	0.0008 (16)
C38	0.087 (2)	0.090 (2)	0.089 (2)	−0.0096 (17)	−0.0133 (16)	0.0021 (17)
C33	0.150 (4)	0.257 (7)	0.110 (4)	−0.012 (4)	0.023 (3)	−0.004 (4)
C32	0.139 (3)	0.114 (3)	0.094 (3)	0.012 (2)	0.012 (2)	0.031 (2)
C35	0.124 (3)	0.124 (3)	0.094 (3)	−0.001 (2)	−0.003 (2)	−0.019 (2)
C39	0.103 (3)	0.100 (3)	0.123 (3)	−0.025 (2)	−0.024 (2)	0.009 (2)
C37	0.194 (6)	0.329 (10)	0.123 (4)	−0.008 (6)	0.002 (4)	0.032 (5)
C40	0.099 (3)	0.133 (3)	0.194 (4)	−0.035 (2)	−0.046 (3)	0.010 (3)
C29	0.100 (3)	0.137 (3)	0.117 (3)	−0.047 (2)	0.016 (2)	0.016 (3)
C36	0.151 (4)	0.195 (5)	0.115 (3)	−0.008 (3)	−0.009 (3)	0.024 (3)
C34	0.215 (6)	0.300 (9)	0.107 (4)	−0.033 (6)	0.025 (4)	−0.003 (5)
C31	0.376 (11)	0.171 (6)	0.127 (4)	−0.023 (6)	−0.033 (5)	0.048 (4)
C30	0.170 (5)	0.214 (6)	0.151 (4)	−0.078 (5)	0.037 (4)	0.046 (4)

### Geometric parameters (Å, º)

**Table:**

O6—C13	1.378 (3)	C21—H21A	0.9700
O6—C38	1.445 (3)	C21—H21B	0.9700
O4—C20	1.384 (3)	C19—C18	1.395 (4)
O4—C32	1.432 (4)	C18—C17	1.365 (4)
O5—C1	1.379 (3)	C18—H18	0.9300
O5—C35	1.411 (4)	C3—C4	1.373 (4)
O3—C23	1.389 (3)	C3—H3	0.9300
O3—C29	1.429 (4)	C5—C4	1.388 (4)
O2—C42	1.203 (3)	C5—H5	0.9300
C1—C6	1.386 (4)	C16—C17	1.363 (4)
C1—C2	1.394 (3)	C16—H16	0.9300
C11—C12	1.384 (3)	C17—H17	0.9300
C11—C10	1.391 (3)	C4—H4	0.9300
C11—H11	0.9300	C41—H41	0.9300
C10—C9	1.389 (3)	C38—C39	1.477 (4)
C10—C42	1.458 (4)	C38—H38A	0.9700
C12—C13	1.401 (3)	C38—H38B	0.9700
C12—C14	1.514 (3)	C33—C34	1.418 (6)
C26—C25	1.384 (3)	C33—C32	1.449 (6)
C26—C27	1.388 (3)	C33—H33A	0.9700
C26—C41	1.459 (4)	C33—H33B	0.9700
C25—C24	1.388 (4)	C32—H32A	0.9700
C25—H25	0.9300	C32—H32B	0.9700
C24—C23	1.395 (3)	C35—C36	1.508 (6)
C24—C28	1.515 (3)	C35—H35A	0.9700
C9—C8	1.382 (3)	C35—H35B	0.9700
C9—H9	0.9300	C39—C40	1.516 (5)
C13—C8	1.390 (3)	C39—H39A	0.9700
C23—C22	1.395 (3)	C39—H39B	0.9700
C6—C5	1.382 (4)	C37—C36	1.430 (7)
C6—C7	1.507 (3)	C37—H37A	0.9600
C27—C22	1.386 (3)	C37—H37B	0.9600
C27—H27	0.9300	C37—H37C	0.9600
O1—C41	1.205 (3)	C40—H40A	0.9600
C20—C19	1.386 (4)	C40—H40B	0.9600
C20—C15	1.393 (3)	C40—H40C	0.9600
C2—C3	1.377 (4)	C29—C30	1.486 (6)
C2—C28	1.510 (4)	C29—H29A	0.9700
C22—C21	1.516 (4)	C29—H29B	0.9700
C8—C7	1.523 (3)	C36—H36A	0.9700
C14—C15	1.510 (4)	C36—H36B	0.9700
C14—H14A	0.9700	C34—H34A	0.9600
C14—H14B	0.9700	C34—H34B	0.9600
C42—H42	0.9300	C34—H34C	0.9600
C15—C16	1.401 (4)	C31—C30	1.377 (8)
C7—H7A	0.9700	C31—H31A	0.9600
C7—H7B	0.9700	C31—H31B	0.9600
C28—H28A	0.9700	C31—H31C	0.9600
C28—H28B	0.9700	C30—H30A	0.9700
C21—C19	1.504 (4)	C30—H30B	0.9700
			
C13—O6—C38	114.1 (2)	C4—C3—H3	119.2
C20—O4—C32	115.9 (2)	C2—C3—H3	119.2
C1—O5—C35	115.4 (2)	C6—C5—C4	120.7 (3)
C23—O3—C29	112.6 (2)	C6—C5—H5	119.6
O5—C1—C6	117.7 (2)	C4—C5—H5	119.6
O5—C1—C2	119.6 (2)	C17—C16—C15	121.1 (3)
C6—C1—C2	122.4 (2)	C17—C16—H16	119.5
C12—C11—C10	121.6 (2)	C15—C16—H16	119.5
C12—C11—H11	119.2	C16—C17—C18	120.6 (3)
C10—C11—H11	119.2	C16—C17—H17	119.7
C9—C10—C11	118.7 (2)	C18—C17—H17	119.7
C9—C10—C42	119.7 (2)	C3—C4—C5	119.5 (3)
C11—C10—C42	121.6 (2)	C3—C4—H4	120.2
C11—C12—C13	117.4 (2)	C5—C4—H4	120.2
C11—C12—C14	119.8 (2)	O1—C41—C26	125.5 (3)
C13—C12—C14	122.7 (2)	O1—C41—H41	117.3
C25—C26—C27	119.0 (2)	C26—C41—H41	117.3
C25—C26—C41	121.0 (2)	O6—C38—C39	114.0 (3)
C27—C26—C41	120.0 (2)	O6—C38—H38A	108.8
C26—C25—C24	121.4 (2)	C39—C38—H38A	108.8
C26—C25—H25	119.3	O6—C38—H38B	108.8
C24—C25—H25	119.3	C39—C38—H38B	108.8
C25—C24—C23	117.7 (2)	H38A—C38—H38B	107.7
C25—C24—C28	119.6 (2)	C34—C33—C32	116.0 (5)
C23—C24—C28	122.5 (2)	C34—C33—H33A	108.3
C8—C9—C10	121.8 (2)	C32—C33—H33A	108.3
C8—C9—H9	119.1	C34—C33—H33B	108.3
C10—C9—H9	119.1	C32—C33—H33B	108.3
O6—C13—C8	119.7 (2)	H33A—C33—H33B	107.4
O6—C13—C12	117.8 (2)	O4—C32—C33	113.4 (4)
C8—C13—C12	122.4 (2)	O4—C32—H32A	108.9
O3—C23—C24	118.6 (2)	C33—C32—H32A	108.9
O3—C23—C22	118.7 (2)	O4—C32—H32B	108.9
C24—C23—C22	122.6 (2)	C33—C32—H32B	108.9
C5—C6—C1	117.9 (2)	H32A—C32—H32B	107.7
C5—C6—C7	121.4 (3)	O5—C35—C36	112.4 (4)
C1—C6—C7	120.3 (2)	O5—C35—H35A	109.1
C22—C27—C26	122.1 (2)	C36—C35—H35A	109.1
C22—C27—H27	119.0	O5—C35—H35B	109.1
C26—C27—H27	119.0	C36—C35—H35B	109.1
O4—C20—C19	118.3 (2)	H35A—C35—H35B	107.9
O4—C20—C15	118.8 (2)	C38—C39—C40	111.5 (3)
C19—C20—C15	122.6 (2)	C38—C39—H39A	109.3
C3—C2—C1	117.4 (3)	C40—C39—H39A	109.3
C3—C2—C28	121.9 (2)	C38—C39—H39B	109.3
C1—C2—C28	120.0 (2)	C40—C39—H39B	109.3
C27—C22—C23	117.0 (2)	H39A—C39—H39B	108.0
C27—C22—C21	120.6 (2)	C36—C37—H37A	109.5
C23—C22—C21	122.2 (2)	C36—C37—H37B	109.5
C9—C8—C13	117.7 (2)	H37A—C37—H37B	109.5
C9—C8—C7	119.5 (2)	C36—C37—H37C	109.5
C13—C8—C7	122.8 (2)	H37A—C37—H37C	109.5
C15—C14—C12	109.7 (2)	H37B—C37—H37C	109.5
C15—C14—H14A	109.7	C39—C40—H40A	109.5
C12—C14—H14A	109.7	C39—C40—H40B	109.5
C15—C14—H14B	109.7	H40A—C40—H40B	109.5
C12—C14—H14B	109.7	C39—C40—H40C	109.5
H14A—C14—H14B	108.2	H40A—C40—H40C	109.5
O2—C42—C10	125.6 (3)	H40B—C40—H40C	109.5
O2—C42—H42	117.2	O3—C29—C30	109.7 (3)
C10—C42—H42	117.2	O3—C29—H29A	109.7
C20—C15—C16	116.9 (2)	C30—C29—H29A	109.7
C20—C15—C14	120.5 (2)	O3—C29—H29B	109.7
C16—C15—C14	122.0 (2)	C30—C29—H29B	109.7
C6—C7—C8	110.41 (19)	H29A—C29—H29B	108.2
C6—C7—H7A	109.6	C37—C36—C35	114.9 (4)
C8—C7—H7A	109.6	C37—C36—H36A	108.5
C6—C7—H7B	109.6	C35—C36—H36A	108.5
C8—C7—H7B	109.6	C37—C36—H36B	108.5
H7A—C7—H7B	108.1	C35—C36—H36B	108.5
C2—C28—C24	109.6 (2)	H36A—C36—H36B	107.5
C2—C28—H28A	109.8	C33—C34—H34A	109.5
C24—C28—H28A	109.8	C33—C34—H34B	109.5
C2—C28—H28B	109.8	H34A—C34—H34B	109.5
C24—C28—H28B	109.8	C33—C34—H34C	109.5
H28A—C28—H28B	108.2	H34A—C34—H34C	109.5
C19—C21—C22	110.0 (2)	H34B—C34—H34C	109.5
C19—C21—H21A	109.7	C30—C31—H31A	109.5
C22—C21—H21A	109.7	C30—C31—H31B	109.5
C19—C21—H21B	109.7	H31A—C31—H31B	109.5
C22—C21—H21B	109.7	C30—C31—H31C	109.5
H21A—C21—H21B	108.2	H31A—C31—H31C	109.5
C20—C19—C18	117.3 (2)	H31B—C31—H31C	109.5
C20—C19—C21	119.9 (2)	C31—C30—C29	115.9 (6)
C18—C19—C21	122.3 (3)	C31—C30—H30A	108.3
C17—C18—C19	121.0 (3)	C29—C30—H30A	108.3
C17—C18—H18	119.5	C31—C30—H30B	108.3
C19—C18—H18	119.5	C29—C30—H30B	108.3
C4—C3—C2	121.7 (3)	H30A—C30—H30B	107.4
			
C35—O5—C1—C6	108.8 (3)	C11—C12—C14—C15	−53.0 (3)
C35—O5—C1—C2	−77.7 (3)	C13—C12—C14—C15	124.8 (2)
C12—C11—C10—C9	−4.2 (3)	C9—C10—C42—O2	171.8 (3)
C12—C11—C10—C42	175.0 (2)	C11—C10—C42—O2	−7.4 (4)
C10—C11—C12—C13	−1.7 (3)	O4—C20—C15—C16	179.5 (2)
C10—C11—C12—C14	176.2 (2)	C19—C20—C15—C16	−7.2 (4)
C27—C26—C25—C24	−1.5 (4)	O4—C20—C15—C14	−8.6 (3)
C41—C26—C25—C24	−179.0 (2)	C19—C20—C15—C14	164.7 (2)
C26—C25—C24—C23	−2.1 (3)	C12—C14—C15—C20	−63.7 (3)
C26—C25—C24—C28	173.1 (2)	C12—C14—C15—C16	107.8 (3)
C11—C10—C9—C8	5.1 (3)	C5—C6—C7—C8	−106.7 (3)
C42—C10—C9—C8	−174.1 (2)	C1—C6—C7—C8	66.5 (3)
C38—O6—C13—C8	−74.2 (3)	C9—C8—C7—C6	49.8 (3)
C38—O6—C13—C12	109.0 (3)	C13—C8—C7—C6	−127.2 (3)
C11—C12—C13—O6	−176.3 (2)	C3—C2—C28—C24	107.4 (3)
C14—C12—C13—O6	5.9 (3)	C1—C2—C28—C24	−62.3 (3)
C11—C12—C13—C8	7.0 (3)	C25—C24—C28—C2	−49.5 (3)
C14—C12—C13—C8	−170.8 (2)	C23—C24—C28—C2	125.5 (3)
C29—O3—C23—C24	91.3 (3)	C27—C22—C21—C19	46.3 (3)
C29—O3—C23—C22	−92.1 (3)	C23—C22—C21—C19	−129.5 (2)
C25—C24—C23—O3	−178.7 (2)	O4—C20—C19—C18	−179.6 (2)
C28—C24—C23—O3	6.3 (3)	C15—C20—C19—C18	7.1 (4)
C25—C24—C23—C22	4.8 (3)	O4—C20—C19—C21	8.2 (3)
C28—C24—C23—C22	−170.2 (2)	C15—C20—C19—C21	−165.1 (2)
O5—C1—C6—C5	179.5 (2)	C22—C21—C19—C20	65.1 (3)
C2—C1—C6—C5	6.1 (4)	C22—C21—C19—C18	−106.7 (3)
O5—C1—C6—C7	6.0 (3)	C20—C19—C18—C17	−1.9 (4)
C2—C1—C6—C7	−167.3 (2)	C21—C19—C18—C17	170.2 (3)
C25—C26—C27—C22	2.6 (4)	C1—C2—C3—C4	1.6 (4)
C41—C26—C27—C22	−179.9 (2)	C28—C2—C3—C4	−168.3 (3)
C32—O4—C20—C19	108.0 (3)	C1—C6—C5—C4	−0.4 (4)
C32—O4—C20—C15	−78.4 (3)	C7—C6—C5—C4	172.9 (2)
O5—C1—C2—C3	−180.0 (2)	C20—C15—C16—C17	2.2 (4)
C6—C1—C2—C3	−6.7 (4)	C14—C15—C16—C17	−169.6 (2)
O5—C1—C2—C28	−9.9 (4)	C15—C16—C17—C18	2.7 (4)
C6—C1—C2—C28	163.4 (2)	C19—C18—C17—C16	−2.9 (4)
C26—C27—C22—C23	0.0 (3)	C2—C3—C4—C5	3.8 (4)
C26—C27—C22—C21	−176.1 (2)	C6—C5—C4—C3	−4.4 (4)
O3—C23—C22—C27	179.7 (2)	C25—C26—C41—O1	−1.9 (4)
C24—C23—C22—C27	−3.8 (3)	C27—C26—C41—O1	−179.3 (3)
O3—C23—C22—C21	−4.3 (3)	C13—O6—C38—C39	−71.0 (3)
C24—C23—C22—C21	172.2 (2)	C20—O4—C32—C33	−87.1 (4)
C10—C9—C8—C13	−0.1 (3)	C34—C33—C32—O4	−173.9 (5)
C10—C9—C8—C7	−177.2 (2)	C1—O5—C35—C36	−85.5 (4)
O6—C13—C8—C9	177.2 (2)	O6—C38—C39—C40	178.7 (3)
C12—C13—C8—C9	−6.2 (3)	C23—O3—C29—C30	−175.7 (4)
O6—C13—C8—C7	−5.7 (3)	O5—C35—C36—C37	−173.5 (5)
C12—C13—C8—C7	170.9 (2)	O3—C29—C30—C31	73.4 (7)
